# Differences in Knowledge of Breast Cancer Screening Among African American, Arab American, and Latina Women

**Published:** 2010-12-15

**Authors:** Karen Patricia Williams, Athur Mabiso, David Todem, Adnan Hammad, Hiam Hamade, Yolanda Hill-Ashford, Murlisa Robinson-Lockett, Gloria Palamisono, Ruth E. Zambrana

**Affiliations:** Obstetrics, Gynecology, and Reproductive Biology, College of Human Medicine, Michigan State University; Michigan State University, East Lansing, Michigan; Michigan State University, East Lansing, Michigan; Arab Community Center for Economic and Social Services, Dearborn, Michigan; Arab Community Center for Economic and Social Services, Dearborn, Michigan; Detroit Department of Health and Wellness Promotion, Detroit, Michigan; Detroit Department of Health and Wellness Promotion, Detroit, Michigan; Community Center for Health and Social Services, Inc, Detroit, Michigan; University of Maryland, College Park, Maryland

## Abstract

**Introduction:**

We examined differences in knowledge and socioeconomic factors associated with 3 types of breast cancer screening (breast self-examination, clinical breast examination, and mammogram) among African American, Arab, and Latina women.

**Methods:**

Community health workers used a community-based intervention to recruit 341 women (112 Arab, 113 Latina, and 116 African American) in southeastern Michigan to participate in a breast cancer prevention intervention from August through October 2006. Before and after the intervention, women responded to a previously validated 5-item multiple-choice test on breast cancer screening (possible score range: 0 to 5) in their language of preference (English, Spanish, or Arabic). We used generalized estimating equations to analyze data and to account for family-level and individual correlations.

**Results:**

Although African American women knew more about breast cancer screening at the baseline (pretest median scores were 4 for African American, 3 for Arab and 3 for Latina women), all groups significantly increased their knowledge after participating in the breast cancer prevention intervention (posttest median scores were 5 for African American and 4 for Arab and Latina women). Generalized estimating equations models show that Arab and Latina women made the most significant gains in posttest scores (*P* < .001).

**Conclusion:**

Racial/ethnic differences in knowledge of breast cancer screening highlight the need for tailored information on breast cancer screening for African American, Arab, and Latina women to promote adherence to breast cancer screening guidelines.

## Introduction

Despite growing interest in factors associated with cancer-related disparities and interventions to reduce disparities, information is still limited regarding differences in screening practices by geographic location and effective tailored interventions for specific racial/ethnic groups. African American and Latina women have disproportionately higher death rates due to breast cancer yet nationally lower incidence rates than those of their non-Hispanic white counterparts (1). Although screening rates for breast cancer have increased for African American and Latina women, breast cancer cases among these populations are often diagnosed at later stages, which limits treatment options (1,2). Although national data illuminate prevailing disparities, data are not available for women who are categorized as non-Hispanic white but who consider themselves to be of a different racial/ethnic group and are low-income or medically underserved or both. In this study we included Arab women traditionally categorized as non-Hispanic white but representing 10 northern African countries and 12 Persian Gulf countries (3) who are not ethnically identifiable in national databases such as the Behavioral Risk Factor Surveillance System (BRFSS) (4).

As women's screening rates for breast cancer increase nationally, barriers to screening remain for African American, Arab, and Latina women from poor backgrounds. Multiple factors are associated with breast cancer screening rates among these groups of women, including socioeconomic status, health insurance coverage, usual source of care, perceptions and fears about breast cancer, race, ethnicity, age, and knowledge of breast cancer screening (5-8).

A special edition of the Michigan Cancer Behavioral Risk Factor Survey (BRFS) conducted in 2006 (9) disaggregated Arab women from non-Hispanic white women. The survey showed that screening rates for annual mammography and clinical breast examination were 53% (95% confidence interval [CI], ±6%) and 46% (95% CI, ±20%) for African American and Latina women aged 40 years or older, respectively, which were lower than rates for the general Michigan population (54% [95% CI, ±4%]) and for Arab women (68% [95% CI, ±24%]). Nationally, the 2005 BRFSS indicated that recent mammography screening rates for African American women aged 40 years or older were approximately 67% and for Latinas aged 40 years or older were approximately 65%, comparable to rates for non-Hispanic white women (63%). Mammogram screening data serve as a proxy for adherence to guidelines that recommend yearly mammograms for women aged 40 or older (10). However, the combination of mammography screening and clinical breast examination would give a more accurate picture of current breast cancer screening behaviors overall; guidelines also recommend clinical breast examinations every 3 years for women in their 20s and 30s and every year for women aged 40 years or older (10).

We studied differences in knowledge of and socioeconomic factors associated with breast cancer screening and assessed baseline and postintervention test scores on the knowledge of breast cancer screening practices among African American, Arab, and Latina women who participated in the Kin Keeper Cancer Prevention Intervention, a family-focused educational intervention for women (11). We hypothesized that, whereas all 3 population groups should be targeted for the educational intervention, Arab and Latina women have lower socioeconomic status and lower levels of knowledge about screening for breast cancer than do African American women. These racial/ethnic differences are important for the design of tailored interventions because they are likely to affect the screening behaviors and, ultimately, the breast health outcomes among these groups of women.

## Methods

### Data collection and sample

The Michigan State University institutional review board approved this study. The locations of the community-based study were southeastern Michigan, in the cities of Detroit and Dearborn. Detroit is the largest city in Michigan and the 11th most-populated city in the United States. Approximately 80% of the residents are African American and 5% are Latino/a, according to the 2000 US Census. The 2006-2008 US Department of Treasury American Community Survey data for Michigan indicates that the city's median annual household income is $29,526 compared with Dearborn's $44,650. Nearly 33% of the residents in Dearborn are Arab; the city has the largest Arab population outside of the Middle East.

Study participants came from 1 of 3 community-based organizations affiliated with the Detroit Department of Health and Wellness Promotion: 1) Village Health Worker Program, 2) Community Health and Social Services (CHASS), and 3) the Arab Community Center for Economic and Social Services (ACCESS). Although the health department serves all racial/ethnic groups, for the purposes of this study, it was asked to recruit only African American and Latina women through its healthy lifestyle program. CHASS, known for its specialized services to Latinos/as in Detroit, recruited Latinas from the REACH Detroit Partnership (a diabetes prevention and complications program). ACCESS, which is located in Dearborn, recruited Arab women from its healthy lifestyle program. The organizations had credibility in the community and employed community health workers (CHWs). We used the Kin Keeper model to recruit women and deliver breast cancer education in the homes of a family member (the kin keeper) (11-13).

### Kin Keeper Cancer Prevention Intervention

The Kin Keeper Cancer Prevention Intervention is community-based; it uses CHWs to educate groups of female family members about breast or cervical cancer or both (11-13). In 2 home visits, the CHWs use breast models and other educational visual aids to teach participants about breast cancer, early detection, clinical screenings, and breast self-examination. At each education session, the participants are given pretests and posttests designed to assess their knowledge of breast cancer screening. Respondents have the option to hear and see test questions and receive the educational intervention in their language of preference (English, Arabic, or Spanish) (14). As part of the Kin Keeper model, CHWs read the questions aloud while participants follow and write their responses. At the end of the second home visit, the CHW works with each participant individually to set up a personal action plan that allows her to set screening goals for a 12-month follow-up visit. For the purposes of this article, we focus only on the pretest and posttest results from the first 3 months (August through October 2006).

### Recruitment

This model has a unique recruitment method: CHWs recruit clients/kin keepers and kin keepers recruit female family members. It begins with cross-training CHWs (from their respective noncancer-related public health programs) in the basics of breast and cervical cancer prevention and control and recruitment of clients into the study (11,13). After 20 hours of training, CHWs ask clients from their public health programs to participate in the research project. The recruitment phase of this study was 3 months. Pretests and posttests (12) were administered by CHWs (7 from each racial/ethnic group) to 104 families, comprising 341 women (116 African Americans, 113 Latinas, and 112 Arabs). Clients had to be aged 18 or older, self-identify as belonging to 1 of the 3 racial/ethnic groups and have blood parents and both sets of blood grandparents in the same race/ethnicity, and be willing to gather 3 to 4 of their adult female family members (in any combination — mother, sisters, grandmothers, aunts, daughters) for 2 home-based educational sessions delivered by the CHW. Because the CHW had an established relationship of trust with clients, recruiting clients into the study was not difficult — all eligible clients who were asked to participate in the study consented to become kin keepers. Although other family members were free to hear the education, they were not considered part of the research project. Kin keepers also helped CHWs to locate family members who were part of the study for the follow-up visit when necessary (11). All participants (kin keepers and family) answered questions about their knowledge of breast cancer screening in a familiar environment — the home.

### Home visits

Each family unit received 2 home visits. At the first home visit, participants signed informed consent forms and completed pre-intervention (baseline) sociodemographic forms and a 16-item assessment of breast cancer literacy. Both the sociodemographic and the cancer literacy assessments were administered orally; participants followed as the CHW read in the preferred language. Latina and Arab CHWs were bilingual and at some visits had to read in both English and the preferred language, which allowed us to measure participants' actual knowledge about screening for breast cancer regardless of their ability to read and comprehend the assessment items. From the second home visits, 333 women were retained (114 African Americans, 112 Latinas, and 107 Arabs), resulting in an overall sample retention rate of approximately 98%.

The 16-item assessment tool for breast cancer literacy has 3 domains: 1) cancer awareness, 2) knowledge and screening, and 3) prevention and control. It uses a multiple-choice and true-or-false format, and has been validated (English: Cronbach α, 0.99; Spanish: Cronbach α, 0.99; and Arabic: Cronbach α, 0.81) (14). For the purposes of this study, we focused only on the knowledge and screening domain, which consists of 5 items (Cronbach α, 0.78) (15). The 5 items are specific to knowledge of breast cancer screening (Appendix). In addition, we analyzed variables from the sociodemographic questionnaire.

**Figure 1:**
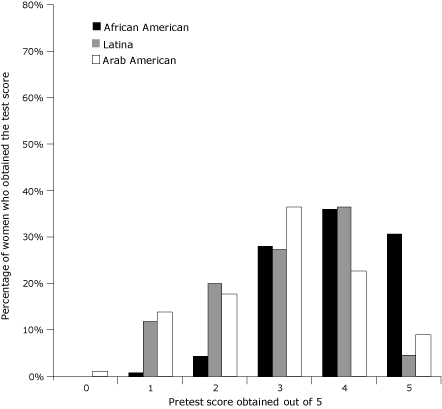
Distribution of pretest scores by race/ethnicity in the Kin Keeper Cancer Prevention Intervention, Dearborn and Detroit, Michigan, August through October 2006. The pretest evaluated baseline knowledge of breast cancer screening methods. The highest possible score on the pretest was 5.

**Table:** 

**Race/ethnicity **	**Percentage of Women Who Obtained the Pretest Score**					

	**0**	**1**	**2**	**3**	**4**	**5**
African American	0	1	4	28	36	31
Latina	0	12	20	27	36	5
Arab	1	14	18	36	23	9

After completing the intervention pretest to assess breast cancer literacy, participants received the education followed by a posttest. The home visit lasted 1.5 to 2 hours. CHWs who needed to speak in 2 languages took longer. At the end of the first home visit, the second home visit was scheduled for 1 to 3 weeks later. At the second home visit, the second posttest was administered before the second educational session. During the second educational session, CHWs cleared up myths, answered questions, and reviewed basic points. Then they administered a third posttest and worked with participants to complete a personal action plan.

### Outcome measures

We analyzed 5 binary outcome variables in this study, each corresponding to a response on the knowledge and screening domain of the assessment tool for breast cancer literacy (1 if correct and 0 if incorrect). We used baseline and postintervention responses (ie, repeated measures) to compare racial/ethnic differences in knowledge of breast cancer screening, which allowed us also to assess changes in knowledge of breast cancer screening for each race/ethnicity.

### Statistical analysis

We graphed the distributions of the pretest and posttest scores and computed descriptive statistics for the whole sample and by racial/ethnic group. Sociodemographic characteristics were analyzed by race/ethnicity. Two-sided χ^2^ and Fisher exact tests were performed to test for categorical association. For these preliminary analyses, familial association was ignored. We used SAS version 9.1 (SAS Institute, Inc, Cary, North Carolina) to perform all statistical tests and modeling. Statistical significance was set at *P* < .05.

To analyze differences in women's knowledge of breast cancer screening by race/ethnicity and across time, we considered generalized estimating equations (GEE) models (16-19) accounting for the familial associations and for participant associations (over time). We anticipated that our data would be correlated because women were recruited from the same families and longitudinal measurements were recorded on each participant. It is well-established that ignoring these associations (for example, by fitting a classical independence logistic regression model) is likely to yield incorrect standard errors of model estimates. The GEE method adjusts these standard errors by using the so-called robust sandwich estimator that corrects for any misspecification of the true underlying correlation (16-19).

The probability of answering each question correctly was modeled separately, controlling for age, income, highest level of education attained, marital status, employment status, and health insurance status. The basic GEE regression model for the binary outcome w*_kfij_* is given by the following equation:

Equation

log(pr(wkfij=1)1−pr(wkfij=1))=βjk+γCkfij

,

[Description of this equation: Logarithm to base 10 of the product of the ratio of the probability that parameter w sub kfij equals 1, to the difference between 1 and the probability that parameter w sub kfij equals 1. This quantity equals the sum of beta sub jk plus gamma cap C sub kfij.]

Let w*_kfij_* be the binary variable defining whether the question under consideration is answered correctly (w*_kfij_* = 1) or not (w*_kfij_* = 0), for woman *i* of family *f *of race *k* at time point *j *(*j* = 1 for baseline test and *j* = 2 for postintervention test). Parameter *β_jk_* represents the log odds of answering correctly a question at time point *j* for race *k*, adjusted for covariates in the design vector *C_kfij_*. The standard error of the estimate of *β_jk_* is typically computed from the sandwich estimator of the variance-covariance matrix of the parameter vector estimate, which takes into account the longitudinal nature of the binary outcomes and the familial clustering.

## Results

**Figure 2:**
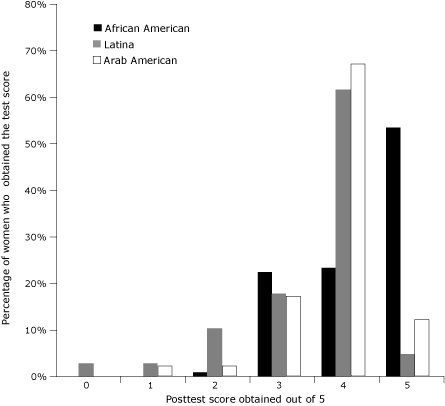
Distribution of posttest scores attained by race/ethnicity in the Kin Keeper Breast Cancer Prevention Intervention, Dearborn and Detroit, Michigan, August through October, 2006. The posttest evaluated knowledge of breast cancer screening methods after the intervention. The highest possible score on the posttest was 5

Sociodemographic characteristics differed significantly by race/ethnicity (Table 1). African American women had higher levels of education, employment, access to health insurance, and income. Approximately 53% of Arab women and 41% of Latina women had not completed high school education or obtained a general equivalency diploma, compared with approximately 3% of African American women (*P* < .001). Overall, 61% of the women were aged 40 years or older, the age category of women recommended for yearly mammography screening (10). Scores on baseline knowledge test (Figure 1) and postintervention tests (Figure 2) were compared by race/ethnicity. At baseline, African American women had higher knowledge scores; more than 30% obtained a perfect pretest score compared with 9% for Arab women and 5% for Latina women. Mean pretest scores were 3.91 of 5 (SD, 0.92) for African Americans, 3.02 (SD, 1.12) for Latinas, and 2.92 (SD, 1.18) for Arabs. Posttest scores for Latina and Arab women increased significantly (*P* < .001), as depicted by distribution, which is relatively skewed compared with the pretest distribution in Figure 1. Posttest median scores were 5 for African American women and 4 for Latina and Arab women. The percentage of women in each racial/ethnic group that answered each question correctly varied (Table 2). Question 4, which asked the women to differentiate between types of screening test (self-examination, clinical examination, and mammography) proved to be the most difficult (Table 2) for all racial/ethnic groups.

**Table:** 

**Race/ethnicity **	**Percentage of Women Who Obtained the Posttest Score**					

	**0**	**1**	**2**	**3**	**4**	**5**
African American	0	0	1	22	23	53
Latina	3	3	10	18	62	5
Arab	0	2	2	17	67	12

In the pretest, Arab women had lower odds of correctly answering the first question (who does a breast self-examination?) than did African American women (odds ratio [OR], 0.27; 95% CI, 0.08-0.89), adjusted for other sociodemographic variables (Table 3). Similarly, for the second question (who does a clinical breast examination?), and the fourth question, which tested the ability to distinguish screening types, Arab women were less likely to answer correctly in the pretest. However, for the third question (who does a mammogram?) and the last question, which distinguished who performs each screening type, the odds of answering the question correctly in the pretest were lower for Arab women but not significantly.

For Latinas, pretest results were somewhat similar to those of Arabs. The GEE-based analysis showed that Latinas had lower odds of correctly answering each question in the pretest than African Americans, except for questions 3, 4, and 5, in which the GEE-based odds were not significant. Latinas had significantly lower odds of knowing the differences between breast self-examination, clinical breast examination, and mammogram than did African Americans.

Posttest GEE estimates show that, compared with African American women, Arab women had lower odds of correctly answering questions 1 and 4, whereas Latina women had significantly lower odds of correctly answering question 4.

## Discussion

To our knowledge this is the first study to empirically compare knowledge of breast cancer screening types among African American, Arab, and Latina women. It yielded 3 major findings: 1) knowledge of breast cancer screening practices was highly associated with race/ethnicity; 2) Arabs and Latinas had similar patterns of knowledge and lower levels of education and insurance coverage compared with African American women; and 3) knowledge of breast cancer screening significantly increased for Latinas and Arabs after community-based intervention, as evidenced by posttest scores.

Overall, the results show that, controlling for sociodemographic characteristics, Arab and Latina women had lower pretest and posttest scores for breast cancer screening knowledge compared with African American women, despite significantly improving their scores in the posttest. Respondents did not fully understand the differences in breast cancer screening types.

African American women's socioeconomic characteristics were highly associated with their higher knowledge levels. Overall, women with higher education levels had higher baseline knowledge of breast cancer screening and reported higher levels of breast cancer screening regardless of race/ethnicity. This finding is consistent with the results of previous studies that show education and access to health care are major predictors of cancer screening knowledge and practices (20,21).

The higher scores on knowledge of breast cancer screening among African American women may also be a result of benefits they received from earlier cancer disparities research that focused on African Americans and breast cancer screening awareness campaigns conducted in English. For non–English-speaking Latinas and Arabs, breast cancer awareness campaigns using Spanish and Arabic messages and materials (12,22) have been made available more recently. Also, popular breast cancer websites do not offer educational materials in Arabic. This disparity might explain the lower baseline scores among Arab and Latina women. More than half the Arab (54%) and Latina (54%) women reported not having health insurance. Other factors — low educational levels and limited English language proficiency, combined with low health literacy, limited availability of linguistically and culturally appropriate materials on breast cancer, and limited experience with the health care system — are strongly associated with knowledge barriers regarding breast cancer screening (23).

Irrespective of a woman's education level, posttest scores increased among the 3 groups; Latina and Arab women achieved the largest gains in knowledge of breast cancer screening. These findings demonstrate that information on breast cancer screening can be effectively provided when interventions address barriers. The Kin Keeper intervention mitigated 3 barriers: low education levels, literacy, and limited English language proficiency (24). If the intervention had not been administered orally and without language preference, 76 (68%) of 112 Arab women would have been missed. Oral administration of the questionnaire permitted the researchers to measure what respondents knew regardless of the respondents' ability to read Arabic or English. Translated materials that are linguistically and culturally appropriate and at an appropriate literacy level (14) increase the effectiveness of cancer screening interventions for women of specific ethnicities.

For Arab women, community-based interventions are necessary to reduce disparities, given that they are more likely than non-Hispanic white women to have irregular screenings and cancer that is detected at later stages (24). Language, access to health care, and geographic residence have also been found to adversely affect Latinas' screening practices (25,26).

Although CHWs completed the same training (27) and had the same basic information regarding specifics of breast cancer education, they had flexibility to tailor their home education sessions. The Latina and Arab CHWs found this especially helpful when talking with older participants, who did not speak or understood very little English, or women who did not socialize much and had various cultural or educationally shaped perceptions of cancer (28). The CHWs' knowledge of their respondent population, and the CHWs' sensitivity to women's perceptions, cultural and language nuances, and questions informed the educational intervention to ensure that information was transmitted in the context of women's lives. The posttest scores suggest that the intervention was effective. Data were not collected on whether racial/ethnic concordance of CHW with respondent increased participation or learning; it is an area for future inquiry.

Methodologic cautions are warranted in terms of instrument and identification of populations. As observed, future research needs to include definitions of medical screening procedures for women to ensure their understanding of survey questions being administered, particularly for low-income women with limited proficiency in English. A community-based study, by design, represents the unique needs of a particular geographic area and designated population groups. Therefore, the results are not meant to be applicable to the general US population. Other limitations include lack of longitudinal data to report knowledge retention and length of residency status of Latinas and Arabs who selected a language other than English.

The inclusion of Arab women presented several challenges. Although the Arab world comprises various countries, including 10 in Africa (3), Arabs have traditionally been classified as white and combined with other non-Hispanic whites and nonmultiracial groups in survey data. Therefore, their unique health status is masked. Furthermore, cancer is not readily discussed among Arab groups because of its severity and the perception that all cancer is hereditary (29). The similar socioeconomic characteristics of Arab and Latina women were predictive of lower baseline knowledge levels, suggesting that low education levels and lack of health insurance coverage are the most powerful predictors of knowledge of breast cancer screening regardless of ethnicity.

Because of the complexity of cancer disparities, education interventions must be developed that are appropriate to the linguistic, health literacy, and cultural needs of participants. Population-specific materials need to be administered in conjunction with community-based participants such as CHWs. Increasing women's knowledge about breast cancer screening is an important first step, but moving women in the direction of adherence to breast cancer screening guidelines and assuring their access to health care services would reduce disparities in breast cancer death and illness.

## Figures and Tables

**Table 1:** Sociodemographic Characteristics of Women Participating in the Kin Keeper Breast Cancer Prevention Intervention, Dearborn and Detroit, Michigan, August-October 2006^a^

Characteristics	African American, No. (%) **(n = 116)**	Arab American, No. (%) (n = 112)	Latina, No. (%) (n = 113)	Total, No. (%) (N = 341)	*P* Value^b^
**Age, y**					
18-39	45 (39)	34 (30)	52 (46)	131 (38)	.03
40-49	23 (20)	38 (34)	24 (21)	85 (25)	
≥50	48 (41)	40 (36)	36 (32)	124 (36)	
**Annual income, $**					
I would prefer not to answer	9 (8)	30 (27)	36 (32)	75 (22)	<.001
<10,000	7 (6)	31 (28)	19 (17)	57 (17)	
10,000-19,999	36 (31)	17 (15)	36 (32)	89 (26)	
20,000-39,999	47 (40)	25 (22)	13 (12)	85 (25)	
≥40,000	17 (15)	9 (8)	9 (8)	35 (10)	
**Education**					
Some college or higher	61 (53)	26 (23)	33 (30)	120 (34)	<.001
High school graduate or GED	52 (45)	27 (24)	33 (29)	112 (33)	
Less than high school or GED	3 (32)	59 (53)	46 (41)	108 (33)	
**Marital status**					
Married	29 (25)	75 (67)	73 (65)	177 (52)	<.001
Widowed/separated/divorced	42 (36)	24 (22)	26 (23)	92 (27)	
Single/never married	43 (37)	10 (9)	13 (12)	66 (19)	
**Employment status**					
Full-time/self-employed	75 (65)	16 (14)	46 (41)	137 (40)	<.001
Part-time	10 (9)	17 (15)	19 (17)	46 (13)	
Unemployed	27 (23)	73 (65)	14 (12)	114 (33)	
Retired/not working because of disability	4 (3)	4 (4)	33 (29)	41 (12)	
**Health insurance**					
Have health insurance	110 (95)	46 (41)	51 (45)	207 (61)	<.001
Do not have health insurance	6 (5)	60 (54)	61 (54)	127 (37)	
**Language of instrument**					
English	116 (100)	36 (32)	28 (25)	180 (53)	NA
Spanish	0	0	85 (75)	85 (25)	
Arabic	0	76 (68)	0	76 (22)	

Abbreviation: GED, general equivalency diploma; NA, not applicable.

a >Percentages may not total 100% because of rounding.

b The χ^2^ statistic was used to calculate *P* values.

**Table 2:** Baseline Knowledge of Breast Cancer Screening Methods by Race/Ethnicity, Kin Keeper Breast Cancer Prevention Intervention, Dearborn and Detroit, Michigan, August-October 2006^a^^,^ b

Pretest Questions	African American, No. (%) (n = 116)	Arab, No. (%) (n = 112)	Latina, No. (%) (n = 113)	Total, No. (%) N = 341	*P* Value^c^
**Question 1: Who does a breast self-examination?**					
Incorrect	7 (6)	32 (29)	38 (34)	77 (23)	<.001
Correct	109 (94)	77 (71)	74 (66)	260 (77)	
**Question 2: Who does a clinical breast examination?**					
Incorrect	3 (3)	28 (26)	24 (21)	55 (16)	<.001
Correct	113 (97)	81 (74)	88 (79)	282 (84)	
**Question 3: Who does a mammogram?**					
Incorrect	10 (9)	27 (26)	34 (31)	71 (21)	<.001
Correct	106 (91)	79 (74)	77 (69)	262 (79)	
**Question 4: Which of these commonly used screening practices are the same?**					
Incorrect	72 (63)	94 (86)	92 (82)	258 (77)	<.001
Correct	42 (37)	15 (14)	20 (18)	77 (23)	
**Question 5: Which of these statements is true?**					
Incorrect	32 (28)	46 (44)	35 (32)	113 (34)	.04
Correct	83 (72)	59 (56)	76 (68)	218 (66)	

a Percentages may not total 100% because of rounding.

b Answer choices are given in the Appendix.

c The 2-sided Fisher exact χ^2^ statistic was used to calculate *P* values.

**Table 3:** Racial/Ethnic Differences in the Probabilities of Answering Each Pretest and Posttest Question Correctly, Kin Keeper Breast Cancer Prevention Intervention, Dearborn and Detroit, Michigan, August-October 2006^a^^,^ b

Questions (No. of Answers)	Arab American		Latina	

	Pretest AOR (95% CI)^a^	Posttest AOR (95% CI)^a^	Pretest AOR (95%CI)	Posttest AOR (95% CI)
Question 1: Who does a breast self-examination? (n = 324)	0.27 (0.08-0.89)	0.78 (0.03-3.10)	0.25 (0.08-0.74)	0.56 (0.19-1.69)
Question 2: Who does a clinical breast examination? (n = 324)	0.11 (0.02-0.48)	0.52 (0.15-1.75)	0.14 (0.04-0.54)	0.39 (0.13-1.18)
Question 3: Who does a mammogram? (n = 320)	0.49 (0.17-1.43)	0.93 (0.31-2.73)	0.40 (0.16-1.01)	0.98 (0.37-2.58)
Question 4: Which of these commonly used screening practices are the same? (n = 322)	0.34 (0.12-0.92)	0.22 (0.09-0.56)	0.37 (0.13-1.00)	0.11 (0.04-0.30)
Question 5: Which of these statements is true? (n = 320)	0.43 (0.16-1.17)	1.95 (0.70-5.45)	0.69 (0.29-1.64)	1.40 (0.58-3.36)

Abbreviations: AOR, adjusted odds ratio, CI, confidence interval.

a Adjusted odds ratios were simultaneously adjusted for age, income, highest level of education attained, marital status, employment status, and health insurance status. African American women are the reference group.

b Answer choices are given in the Appendix.

## Appendix. Five Questions on Knowledge of Types of Screening for Breast Cancer

**Question 1: Who does a breast self-examination?**

*A woman in her home every month.*

A health care provider in a clinic or doctor's office, once a year.

An X-ray technician, once a year.

**Question 2: Who does a clinical breast examination?**

A woman in her home every month.

*A health care provider in a clinic or doctor's office, once a year.*

An X-ray technician, once a year.

**Question 3: Who does a mammogram?**

A woman in her home every month.

A health care provider in a clinic or doctor's office, once a year.

*An X-ray technician, once a year.*

**Question 4: Which of these commonly used screening practices are the same?**

Breast self-examination and clinical breast examination.

Clinical breast examination and mammogram.

Mammogram and breast self-examination.

All are the same.

*None are the same.*

**Question 5: Which of these statements is true?**

Breast self-examinations can be done monthly by all women.

Clinical breast examinations can be done yearly by a health care provider.

Mammograms can be done yearly beginning at age 40, by an X-ray technician.

None of these statements are true.

*All of these statements are true.*

Note: The correct responses are italicized.
